# Hierarchical Self-assembly of Well-Defined Louver-Like P-Doped Carbon Nitride Nanowire Arrays with Highly Efficient Hydrogen Evolution

**DOI:** 10.1007/s40820-020-0399-1

**Published:** 2020-02-17

**Authors:** Bo Li, Yuan Si, Qian Fang, Ying Shi, Wei-Qing Huang, Wangyu Hu, Anlian Pan, Xiaoxing Fan, Gui-Fang Huang

**Affiliations:** 1 Department of Applied Physics, College of Physics and Electronics, and College of Materials Science and Engineering, and State Key Laboratory of Chemo/Biosensing and Chemometrics, College of Chemistry and Chemical Engineering, Hunan University, Changsha, 410082 People’s Republic of China; 2 Department of Physics and Tsinghua-Foxconn Nanotechnology Research Center, Tsinghua University, Beijing, 100084 People’s Republic of China; 3 College of Physics, Liaoning University, Shenyang, 110036 People’s Republic of China

**Keywords:** Self-assembly, Carbon nitride, P-doped, Nanowire arrays, Hydrogen evolution

## Abstract

Self-assembled nanostructure arrays integrating the advantages of the intrinsic characters of nanostructure as well as the array stability are appealing in advanced materials. However, the precise bottom-up synthesis of nanostructure arrays without templates or substrates is quite challenging because of the general occurrence of homogeneous nucleation and the difficult manipulation of noncovalent interactions. Herein, we first report the precisely manipulated synthesis of well-defined louver-like P-doped carbon nitride nanowire arrays (L-PCN) via a supramolecular self-assembly method by regulating the noncovalent interactions through hydrogen bond. With this strategy, CN nanowires align in the outer frame with the separation and spatial location achieving ultrastability and outstanding photoelectricity properties. Significantly, this self-assembly L-PCN exhibits a superior visible light-driven hydrogen evolution activity of 1872.9 μmol h^−1^ g^−1^, rendering a ~ 25.6-fold enhancement compared to bulk CN, and high photostability. Moreover, an apparent quantum efficiency of 6.93% is achieved for hydrogen evolution at 420 ± 15 nm. The experimental results and first-principles calculations demonstrate that the remarkable enhancement of photocatalytic activity of L-PCN can be attributed to the synergetic effect of structural topology and dopant. These findings suggest that we are able to design particular hierarchical nanostructures with desirable performance using hydrogen-bond engineering.

**Electronic supplementary material:**

The online version of this article (10.1007/s40820-020-0399-1) contains supplementary material, which is available to authorized users.

## Highlights

- The self-assembled louver-like P-doped carbon nitride (L-PCN) nanowires is firstly constructed.
- L-PCN nanowires show efficient charge transport, as demonstrated by experimental and theoretical approach.
- L-PCN nanowires exhibit significantly boosted HER activity of 1,872.9 μmol h^−1^ g^−1^ (*λ* > 420 nm).

**Electronic supplementary material:**

The online version of this article (10.1007/s40820-020-0399-1) contains supplementary material, which is available to authorized users.

## Graphical abstract

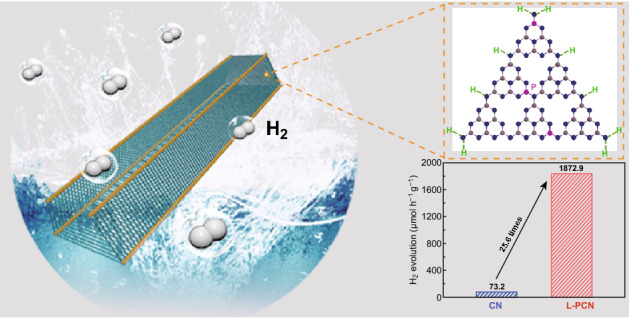

## Introduction

The controlled self-assembly has become a key technology in the development of diversified bottom-up nanostructured materials with desirable properties for numerous applications ranging in electronics, optoelectronic devices to catalysis [1–5]. Recently, research focused on self-assembled nanostructures, such as nanowires [6–8], nanopillars [9], nanotubes [10–13], and nanosheets [14], has led to substantial advances. Major efforts in this field are so far emphasized on the single nanostructure to elucidate structure–property relationships. However, self-assembly of single nanostructure into well-defined nanostructure arrays without templates or substrates has met with little success so far. The reason for the reluctance of researchers to enter this field is given by the difficulties encountered in the design and precise control of the diverse weak noncovalent or covalent bonds interactions, involving in *π*–*π* and charge transfer interactions, hydrogen/halogen bonding, and van der Walls force in the self-assembly process [8, 11, 15]. Even with templates or substrates assisted, the growth of nanostructured arrays is still extremely limited by the self-assembly directions (vertical growth) [4, 6, 16]. Therefore, developing a feasible pathway to releasing the potential of single nanostructures self-assembly into diversified nanostructure arrays is still a great challenge.

Polymeric graphitic carbon nitride (CN) has been becoming a rising star material in recent years due to its extraordinary features and potential applications in the areas of hydrogen evolution, water oxidation, and artificial photosynthesis [17–20]. However, bulk CN obtained by the direct thermal polymerization process generally presents low surface area and fast charge recombination, which significantly restricts its applications [21–23]. To improve the performance of CN, various strategies have been developed and the promising pathways are mainly classified into two categories. One way is the development of various CN nanostructures with optimized physicochemical and optical properties [24, 25]. The other is heteroatom doping to optimizing the electronic and bandgap structures [26–30]. Particularly, much attention has been paid to the self-assembled-specific supramolecular CN nanostructures because of their convenient operation and template-free properties in the synthesis process [10, 31–34]. More exhilaratingly, polymeric CN is generally synthesized via thermal polycondensation of carbon, nitrogen, and hydrogen containing precursors (such as melamine and urea), giving a promising direction of supermolecular self-assembly CN nanostructures based on hydrogen bonding, where the chaotic molecules can be self-assembled because hydrogen bonding has strong direction and saturation [35–39]. Unfortunately, although various CN micro-/nanostructures have been fabricated through thermal polycondensation of the hydrogen containing precursors, the single nanostructures such as microtubes and nanotubes are generally accompanied with serious aggregation and restacking during the practical applications, leading to poor recycling performance [10, 40–42]. Further achievement of hierarchical self-assembly of single CN nanostructure into well-defined nanostructure arrays to impart the CN-based materials with ultrastability as well as highly efficient performance has not yet been reported.

In this work, we first report an approach for the hierarchical self-assembly of well-defined louver-like P-doped CN nanowire arrays (L-PCN) whereby CN nanowires are aligned in the outer frame with the separation and spatial location to retard restacking and improve the hydrogen evolution performance of CN. The hierarchical self-assembly strategy based on the hydrogen bonding interaction between melamine and cyanuric acid develops a precisely well-defined supramolecular precursor via kinetically controlled growth pathway (Fig. S1) under the stirring solution. Moreover, the facile modulation in the formation ratio of the hydrogen bonds by adding appropriate amount of phosphoric acid, which promotes the hydrolysis of melamine to cyanic acid, can realize the quantitative manipulation of the assembly clearance spaces and the exposed degree of active sites in L-PCN. Significantly, the as-prepared L-PCN exhibits a reputably hydrogen evolution activity (1872.9 μmol h^−1^ g^−1^) under visible light irradiation and renders a ~ 25.6-fold enhancement compared to bulk CN. Moreover, an apparent quantum efficiency (AQY) of 6.93% at 420 ± 15 nm is achieved for L-PCN. Characterization of the morphologies as well as specific surface area, charge carrier separation, and transfer efficiency measurements reveals that this outstanding hydrogen evolution performance is attributed to the synergistic effect toward the hierarchical self-assembly louver-like CN nanowire arrays and P-doping.

## Experimental

### Preparation of L-PCN Nanowire Arrays

First, melamine (10 mmol) with different amounts of phosphoric acid (0.5, 1.0, 1.5, and 2.0 mL, the corresponding pH value is 3.5, 2.5, 2, and 1.5) and cyanuric acid (10 mmol) were, respectively, dissolved in deionized water (100 mL) with continuous stirring for 20 min; after melamine and cyanuric acid were completely dissolved, then the melamine solution was slowly poured into the cyanuric acid solution. The mixture solution was centrifuged, washed, and dried at 60 °C. Finally, the resultant solids were heated at 550 °C for 2 h. The catalyst obtained by adding 0.5, 1.0, 1.5, and 2.0 mL of phosphorous acid was named as L-PCN-0.5, L-PCN-1.0, L-PCN-1.5, and L-PCN-2.0, respectively.

### Preparation of Bulk Graphitic CN

Bulk graphitic carbon nitride (CN) was prepared by similar process of L-PCN but without adding phosphorous acid.

### Characterization

The texture properties of the as-prepared samples were examined by X-ray diffraction (XRD, Siemens D-5000 diffractometer) with Cu Kα irradiation source (*λ* = 0.154 nm). Fourier-transform infrared (FT-IR) spectra were recorded on a IR Affnity-1 FTIR spectrometer using KBr pellet as the background. The chemical state of C, N, and P elements was measured through X-ray photoelectron spectroscopy (XPS, PHI Quantera X-ray photoelectron spectrometer) with 300 W Al Kα radiation. Scanning electron microscope (SEM, Hitachi S-4800) and transmission electron microscope (TEM, FEI Tecai F20) were used to observe the morphology of samples. The specific surface areas of the samples were calculated using the Brunauer–Emmett–Teller (BET) method. UV–Vis diffuse reflectance spectra were recorded on a UV-2450 UV–Vis spectrophotometer (Shimadzu Systems, Japan). The photoluminescence (PL) spectra were recorded on a F-2500 fluorescence spectrometer with pulsed xenon discharge lamps upon excitation by incident light of 350 nm at room temperature. Time-resolved fluorescence decay spectra were collected using a streak camera (C10910, Hamamatsu), in which the emission signal was reflected on the streak camera by a mirror.

### Photocatalytic Test

The hydrogen evolution activity was carried out in a Pyrex top-irradiation reaction vessel connected to a closed glass gas system (LabSolar-III AG, Perfectlight Limited, Beijing). A 300 W xenon lamp with a cutoff filter (*λ* ≥ 420 nm) as light source. Generally, photocatalyst (50 mg) loaded with ≈ 1% Pt and methyl alcohol (10 vol%) as sacrificial electron donor were dispersed in aqueous solution (300 mL). The reactant solution was evacuated several times before irradiation to remove the air prior. The temperature of the reaction solution was maintained at 10 °C by a flow of cooling water. The evolved gases were analyzed by gas chromatography (GC7900, Techcomp) equipped with a thermal conductive detector (TCD) for quantification and a 5 A molecular sieve column, using nitrogen as the carrier gas.

The apparent quantum yield (AQY) was measured using a similar experimental setup, only with designated monochromic light (420 nm) to perform the hydrogen evolution. The photointensity was averaged at 20 mW cm^−2^ representative points by PLS-SXE300D photoradiometer, and the irradiation area was approximately 16 cm^2^. The AQY was estimated based on Eq. 1:

1 $$ {\text{AQY}} = \frac{{{\text{number}}\;{\text{of}}\;{\text{evolved}}\;{\text{H}}_{2} \;{\text{molecules}} \times 2}}{{{\text{number}}\;{\text{of}}\;{\text{incident}}\;{\text{photons}}}} \times 100 $$

Photocatalytic degrading organic pollutants of rhodamine B (RhB, 10 mg L^−1^) and bisphenol A (BPA, 10 mg L^−1^) were measured under visible light irradiation (300 W halogen lamp). Typically, 10 mg of catalysts was added into 40 mL model pollutant solution. The suspensions were magnetically stirred in the dark to obtain absorption–desorption equilibrium, and 4 mL of the suspensions was collected at irradiation time intervals and then centrifuged to remove the photocatalyst particles. The concentrations of RhB and BPA were, respectively, analyzed by measuring the absorbance of supernatant at 664 and 275 nm on a TU-1910 UV–Vis spectrophotometer.

### Photoelectrochemical Test

Photoelectrochemical measurements were conducted in a conventional three-electrode cell system using Chen Hua electrochemical station (Shanghai). The fluorine-doped tin oxide (FTO) transparent conductive film glass deposited with samples, Pt wire, and Ag/AgCl electrode were, respectively, used as working electrodes, counterelectrode, and reference electrode. 0.2 M Na_2_SO_4_ aqueous solution (pH = 6.8) was used as the electrolyte. For Mott–Schottky plots, various frequencies of obtained samples were, respectively, at 1.0, 2.3, and 3.1 kHz. For Nyquist plot measurements, the frequency was ranged from 10^−2^ to 10^6^ Hz. The photocurrent response of the photocatalysts as light on and off was measured without bias voltage. Besides, linear sweep voltammetry (LSV) plots for the obtained samples were studied in 0.5 M H_2_SO_4_ aqueous solution with a scan rate of 10 mV s^−1^.

### DFT Calculations

All our calculations are based on density functional theory (DFT) in conjunction with the projector-augmented wave (PAW) potential as implemented by the Vienna ab initio Simulation Package (VASP) [43, 44]. The Perdew–Burke–Ernzerhof generalized gradient approximation (GGA) with the approach of DFT-D2 correction is adopted to correct the weak van der Waals-like interactions [45, 46]. As is known, the GGA functional usually underestimates the band gap of semiconductors. Therefore, the screened hybrid Heyd–Scuseria–Ernzerhof 2006 (HSE06) functional has been employed to obtain accurate electronic structures and optical properties [47, 48]. The cutoff energy for the plane-wave basis set is 500 eV. Geometry optimization is carried out before single-point energy calculation, and the force on the atoms is less than 0.01 eV Å^−1^. The first Brillouin zone is sampled with a Monkhorst–Pack grid of 5 × 5 × 1. All atomic positions were fully relaxed until the force is less than 0.01 eV Å^−1^. We set the criterion for the total energy to be 1.0 × 10^−6^ eV. A vacuum space of 15 Å along the *z* direction is used to avoid artificial interaction.

## Results and Discussion

**Fig. 1:**
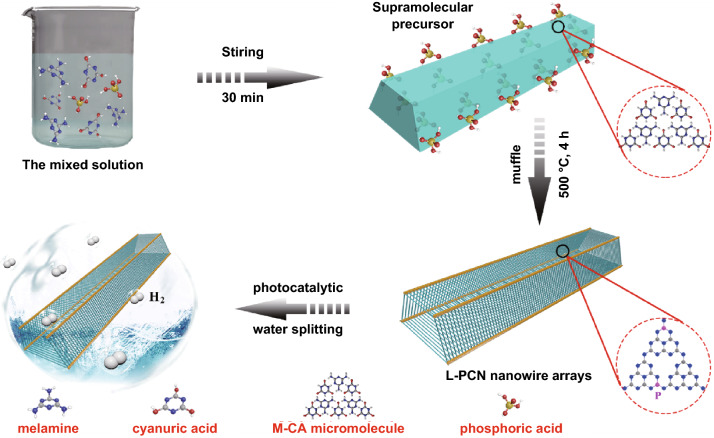
Preparation process of L-PCN nanowire arrays. The quadrangular-like hydrogen-bonded supermolecular precursor is easily prepared by molecular cooperative assembly. Meanwhile, phosphoric acid molecules will be wrapped in or adsorbed on the precursors. After annealing, hierarchical self-assembly of CN nanowire arrays with well-defined louver-like nanostructures (L-CN) is eventually generated through releasing various gas, such as NH_3_, NO/NO_2_, and PH_3_, and the P atoms are successfully doped in the internal framework

**Fig. 2:**
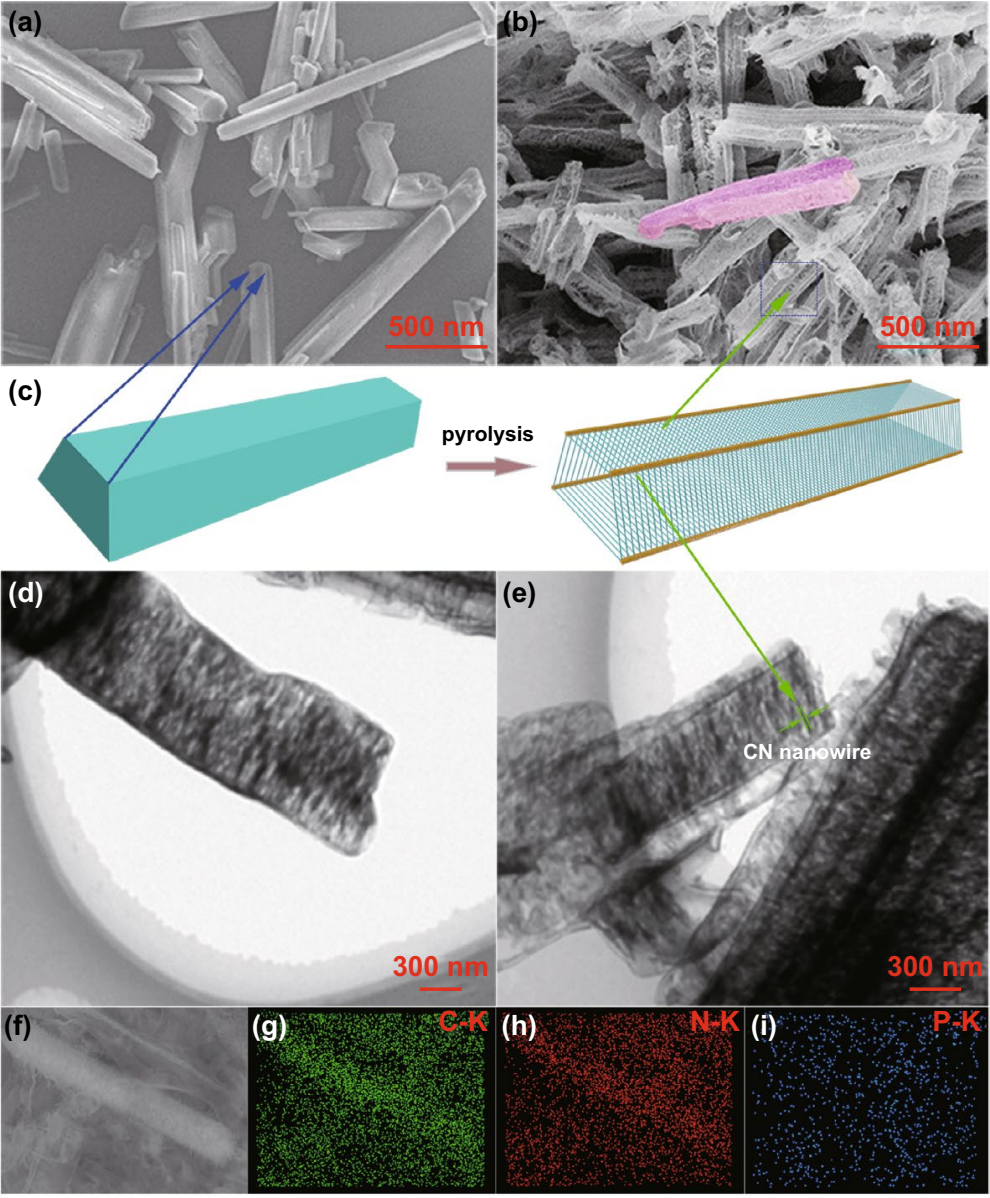
Structural characterization of L-PCN. **a** Typical SEM images of L-PCN-1.0 supramolecular precursor. **b** SEM images of L-PCN-1.0 after thermal treatment with 500 °C, 4 h. **c** Quadrangular and louver model of the supramolecular precursor and L-PCN nanowire arrays. **d**, **e** TEM images of L-PCN-1.0. **f**–**i** EDS elemental mapping images of L-PCN-1.0

The fabrication process of the hierarchical self-assembly of well-defined louver-like P-doped CN nanowire arrays (L-PCN) is shown in Fig. 1. As melamine, cyanuric acid, and phosphorous acid are dissolved in deionized water, phosphoric acid molecules are wrapped in or adsorbed on the immediately formed melamine–cyanuric acid (M–CA) micromolecule assembly, yielding a quadrangular-like P-containing supramolecular precursor. Typical SEM image in Fig. 2a reveals that the obtained quadrangular-like structure has a smooth surface and an edge length of 50–250 nm, indicating the successful molecule self-assembly between melamine and cyanuric acid. After pyrolysis, P atoms squeeze into the CN skeleton and the self-assembled louver-like P-doped CN nanowire arrays (L-PCN) is formed (Fig. 2b, c); the yield of the synthesis is about 10%. The transformation from quadrangular-like precursor to L-PCN nanowire arrays can be mainly ascribed to the release of various gases during pyrolysis, including NH_3_, NO/NO_2_, CO_2_, and PH_3_. It is worth noting that the thermal polymerization of pure M–CA supramolecular precursor (Fig. S2a) only results in irregular bulk CN structure (Fig. S3a). The reason for the distinctly difference morphology between bulk CN and L-PCN lies in the use of phosphoric acid in the reaction solution. The hierarchical self-assembly process based on the hydrogen bonding interaction toward melamine and cyanuric acid can be precisely controlled with phosphorous acid-assisted strategy, since some melamine molecules could be hydrolyzed into cyanuric acid in the presence of phosphorous acid, realizing the quantitative manipulation of the formation of hydrogen bonds. Simultaneously, the phosphorous acid molecules adsorbed on the inside and surface of the quadrangular-like precursor can release a large amount of gas during pyrolysis, which facilitates the separation and spatial location of the self-assembled CN nanowire arrays (Fig. S4). Different volumes of phosphoric acid added from 0.5 to 2.0 mL also lead to smooth quadrangular-like precursors (Fig. S2b–d) and louver-like nanowire arrays (Fig. S3b–d). Moreover, the clearance spaces among CN nanowires in L-PCN change with the increasing in phosphoric acid added, certifying the critical role of phosphoric acid in regulating the hydrogen bonds ratio and the final texture. The hierarchical self-assembly of louver-like nanowire arrays is also clearly observed in TEM images (Fig. 2d, e), and the hierarchical self-assembly of louver-like nanowire arrays with adequate open clearance spaces offers large active sites exposed, enhanced light harvesting, high charge carriers, and mass transport rate. In addition, the elemental mapping illustrated in Fig. 2f–i demonstrates that C, N, and P are distributed uniformly on the surface of L-PCN-1.0 arrays.

**Fig. 3:**
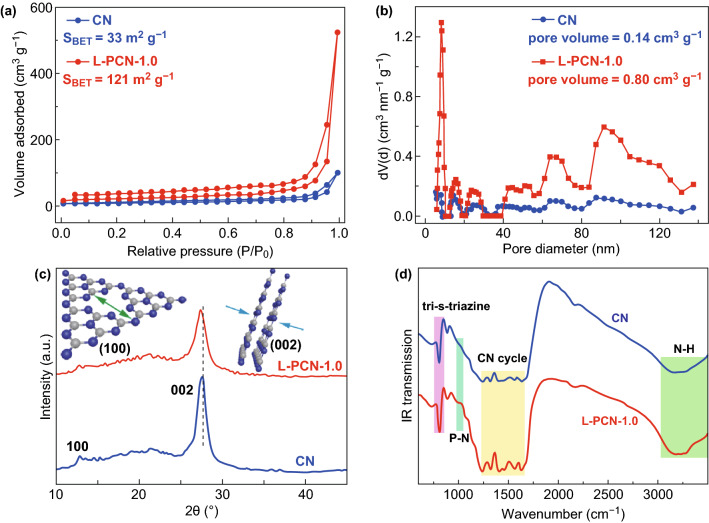
Texture properties and chemical structures of CN and L-PCN. **a** Nitrogen adsorption–desorption isotherms and **b** corresponding pore size distribution curves of CN and L-PCN-1.0. **c**, **d** XRD patterns and FTIR spectra of CN and L-PCN-1.0

The unique hierarchical self-assembly of louver-like CN nanowire arrays motivates us to further study its Brunauer–Emmett–Teller (BET) surface area and pore volume. Thus, N_2_ adsorption–desorption isotherms of bulk CN and L-PCN are investigated. The typical IV adsorption isotherm is obviously observed in Fig. 3a, indicating the characteristic adsorption–desorption hysteresis with well-defined mesopore distribution. The BET surface area of L-PCN-1.0 is calculated to be 121 m^2^ g^−1^ from the linear part of the multipoint plot, which is approximately four times higher than that of bulk CN (ca. 33 m^2^ g^−1^). Compared to bulk CN, the extremely high adsorption capacity in the high relative pressure (P/P_0_ from 0.8 to 1.0) is clearly observed, suggesting the presence of abundant mesopores and/or macropores for L-PCN-1.0 [49, 50], which is well consistent with the SEM and TEM observation. In addition, L-PCN-1.0 also possess larger pore volume (0.80 cm^3^ g^−1^) compared to bulk CN (ca. 0.14 cm^3^ g^−1^). The increased BET surface area and larger pore volume for L-PCN-0.5, L-PCN-1.5, and L-PCN-2.0 are also achieved (Fig. S5 and Table S1) and further confirm that the hierarchical self-assembly of louver-like CN nanowire arrays can effectively promote the kinetics of catalytic reaction by increasing exposed active sites and facilitating mass transfer [51].

The texture properties and chemical structures of pristine CN and L-PCN are characterized by XRD patterns and FTIR spectroscopy. As shown in Fig. 3c, the XRD pattern for L-PCN-1.0 exhibits two characteristic peaks located at 13.2° (100) and 27.4° (002), which are, respectively, associated with in-plane structural packing motif and periodic stacking of layers along the *c*-axis in CN structure [25, 52]. However, it is worth mentioning that the peak at 13.2° and 27.4° is sharply weakened compared to bulk CN, revealing the broken intralayer long-range atomic order and the tri-s-triazine units are stacked in a perpendicular direction to the sheets via *π*–*π* interactions [10]. With increasing phosphoric acid addition, the obtained L-PCN shows a gradually weaker diffraction peaks at 13.2° (Fig. S6a), suggesting the smaller in-planar layer size owing to the “gas etching effect” in L-PCN during thermal condensation. Moreover, the peak at 27.4° slightly shifts to lower 2*θ* degree (Fig. S6b), indicating that P atoms are successfully incorporated into the L-CN framework [53]. For FT-IR spectrum in Fig. 3d, it is clear that L-PCN-1.0 possesses similar characteristic vibration modes compared to bulk CN, indicating that the P-doped hierarchical self-assembly of louver-like nanostructure array does not destroy the fundamental chemical structure of CN. Nevertheless, a pianissimo weak band at 950 cm^−1^ attributed to the P–N stretching mode is found for L-PCN-1.0 [28] and the stretching vibrations band at 808 cm^−1^ for bulk CN shifts to a higher wavenumber of 812 cm^−1^ for L-PCN-1.0 (Fig. S7a), further suggesting that P atoms are successfully incorporated into the triazine ring, which is in agreement with the XRD analysis. Particularly, compared to bulk CN, the gradually stronger band at 3000–3500 cm^−1^ (Fig. S7b) is associated with the stretching vibrations of N–H bond in the marginal amino groups reconfirming the higher hydrogen-bond ratio in the self-assembled L-PCN nanowire arrays.

**Fig. 4:**
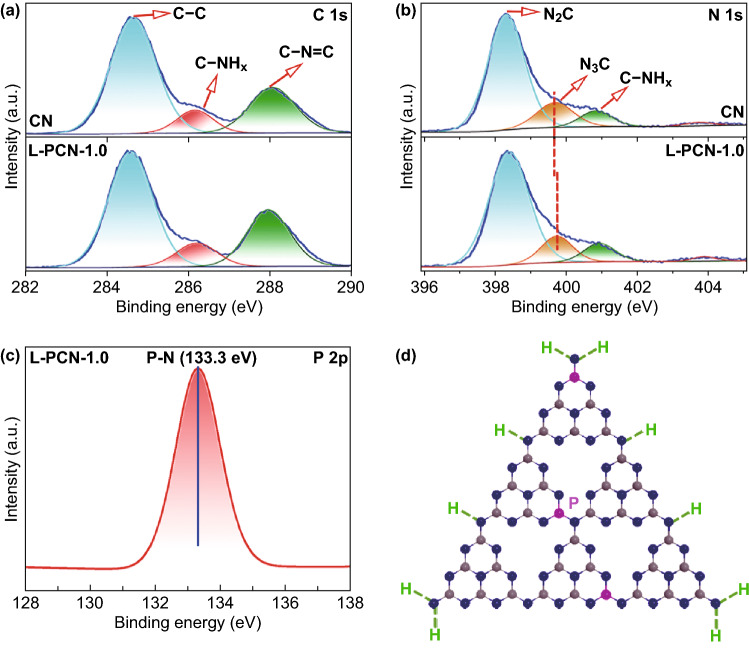
Surface chemical compositions and chemical states characterization of C, N, and P elements for CN and L-PCN. **a**–**c** High resolution of C 1*s*, N 1*s*, and P 2*p* XPS spectra of CN and L-PCN-1.0. **d** Proposed structure of L-PCN (carbon, nitrogen, and phosphorus atoms are, respectively, indicated by gray, blue, and pink spheres in the atomic model). (Color figure online)

XPS measurements are used to ascertain the surface chemical compositions and chemical state of C, N, and P elements in L-PCN. The spectrum in Fig. S8 indicates that bulk CN is mainly composed of C, N, and O elements, while small amount of P can be detected for L-PCN-1.0, suggesting that P atoms are successfully incorporated into the CN framework. The high ratio of C element in CN and L-PCN-1.0 spectrum may be ascribed to the externally contaminated carbon sources, while O element may be attributed to the surface absorbed oxygen-containing species in the samples [49, 54]. The C 1*s* spectra of L-PCN-1.0 can be fitted into three contributions according to the Gaussian rule and, respectively, located at 284.8, 286.4, and 288.1 eV, as shown in Fig. 4a. The peak in 288.1 eV is attributed to *sp*^2^-hybridized carbon atom bonds to the aliphatic amine in the aromatic rings (N–C = N), and the peak at 284.8 and 286.4 eV is, respectively, ascribed to the pure graphitic species (C–C) and C–NH_*x*_ (*x* = 1, 2) on the edges of heptazine units [55]. In particular, the ratio of C–NH_*x*_/C increases from 28.51% for bulk CN to 28.58% for L-PCN-1.0 (Table S2), indicating more hydrogen bonds in L-PCN-1.0. The N 1*s* spectrum for L-PCN-1.0 can be deconvoluted into four peaks at 398.6, 399.9, 401.1, and 404.1 eV, respectively (Fig. 4b). The peak located at 398.6 eV is corresponded to the *sp*^2^-hybridized aromatic in triazine rings [56, 57], whereas the peak located at 399.9 eV is ascribed to the bridging N atoms in N_3_C groups, and the slight shift to higher binding energy compared to CN (399.6 eV) may be ascribed to the changed electron structure in L-PCN-1.0 (Fig. 4b and Table S3) [56]. The peak at 401.1 eV is indexed to the amino functions carrying hydrogen (C–N–H), and the weakness peak in the N 1*s* spectra centered at 404.2 eV is most probably attributed to the positive charge localization in heterocycles [21, 49, 58]. The peak ratio of the amino functions carrying hydrogen is found to be higher than that of bulk CN (Table S2), implying more hydrogen-bond ratio exists in L-PCN-1.0 and in good agreement with the change of C 1*s* (286.4 eV). The survey spectrum of P 2*p* signal is located at around 133.3 eV (Fig. 4c), and the binding energy is higher than P–C bonds (131.5–132.5 eV) and lower than P=N bonds, suggesting that P atoms most probably replace C in triazine rings of CN to form P–N bonds (Fig. 4d) [28, 59–61], consistent with the FTIR spectra results (Fig. 3d). On the other hand, the surface atomic ratio of P element for L-PCN-1.0 is calculated about 0.25% (Table S4). Correspondingly, EDS test demonstrates that the P atomic content is 0.28% (Table S5), indicating the low ratio of P atoms in the CN nanowire arrays matrix.

**Fig. 5:**
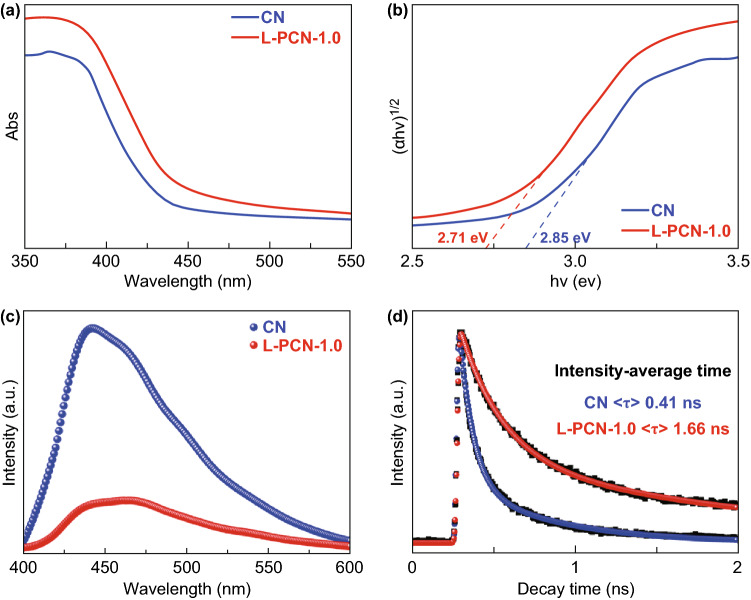
Optical properties characterization of CN and L-PCN. **a** UV–Vis DRS and **b** corresponding band gaps of CN and L-PCN-1.0 determined through the Tauc plots transformed from Kubelka–Munk function. **c** Photoluminescence emission spectra (with 350 nm excitation wavelength at room temperature) of CN and L-PCN-1.0. **d** Time-resolved photoluminescence decay spectra of CN and L-PCN-1.0. These spectra were recorded with the excitation of 400 nm from a picosecond pulsed light-emitting diode at room temperature

The combination of P atom and unique self-assembled L-CN nanowire arrays would alter the optical properties and electronic band structure, which are studied by a combined analysis of optical absorption spectra and fluorescence emission spectra. The UV–Vis diffuse reflectance spectra (DRS) in Fig. 5a show that the absorption edge of L-PCN-1.0 shifts to longer wavelengths compared to bulk CN, indicating that hierarchical self-assembly of CN nanowire arrays can effectively improve the visible light harvesting. Accordingly, the derived electronic band gaps from the Tauc plots reveal the narrowed bandgap energy of L-PCN-1.0 (2.71 eV) compared to 2.85 eV for bulk CN (Fig. 5b) [62]. In addition, the band gaps for L-PCN-0.5, L-PCN-1.5, and L-PCN-2.0 are 2.79, 2.74, and 2.76 eV, respectively (Fig. S9) and further confirm the positive role of P integration and self-assembled L-CN nanowire arrays in enhancing the visible light harvesting ability.

The steady-state photoluminescence (PL) emission spectra of samples are shown in Figs. 5c and S10. Compared to bulk CN, all L-PCN catalysts show lower PL intensity, indicating that hierarchical self-assembled louver-like CN nanowires can efficiently suppress the recombination of photogenerated electron–holes. Moreover, the emission peak red-shifts from about 440 nm for bulk CN to around 480 nm for L-PCN-1.0, which is consistent with DRS spectra. The time-resolved fluorescence decay spectra are also studied to get quantitative information of the photogenerated charge carriers, as shown in Fig. 5d. All spectra are monitored at the wavelength of the maximum emission peak of each product. The recorded emission decay data are analyzed and fitted with a biexponential kinetics model (Eq. 2) [63]:

2 $$ I(t) = A_{1} {\text{e}}^{{ - t/\tau_{1} }} + A_{2} {\text{e}}^{{ - t/\tau_{2} }} $$

which generates two lifetime values, a fast *τ*_1_ and slow *τ*_2_ decay component, and the corresponding amplitudes, *A*_1_ and *A*_2_. The average PL lifetime (*τ*) is deduced by Eq. 3 [60]:

3 $$ (\tau ) = \frac{{A_{1} \tau_{1}^{2} + A_{2} \tau_{2}^{2} }}{{A_{1} \tau_{1} + A_{2} \tau_{2} }} $$

The derived two components of the lifetime and their relative amplitudes are given in Table S5. The resulting average PL lifetime of L-PCN-1.0 (1.66 ns) is 4.04 times longer than the pristine CN (0.41 ns). Specifically, L-PCN-1.0 shows the fast (0.26 ns) for a majority of charge carriers (62.18%) and slow (1.95 ns) with contributions of 37.82%, respectively, while bulk CN exhibits the shortest lifetime (0.08 ns) for a large percentage (75.85%) of charge carriers, suggesting the significantly different emission pathway. In addition, the decreased PL intensity and increased average PL lifetime of L-PCN-0.5, L-PCN-1.5, and L-PCN-2.0 (Figs. S10, S11 and Table S6) have also been achieved, suggesting that the constructed louver-like CN nanowires with P doping are synergistically lengthened the lifetimes of charge carriers.

**Fig. 6:**
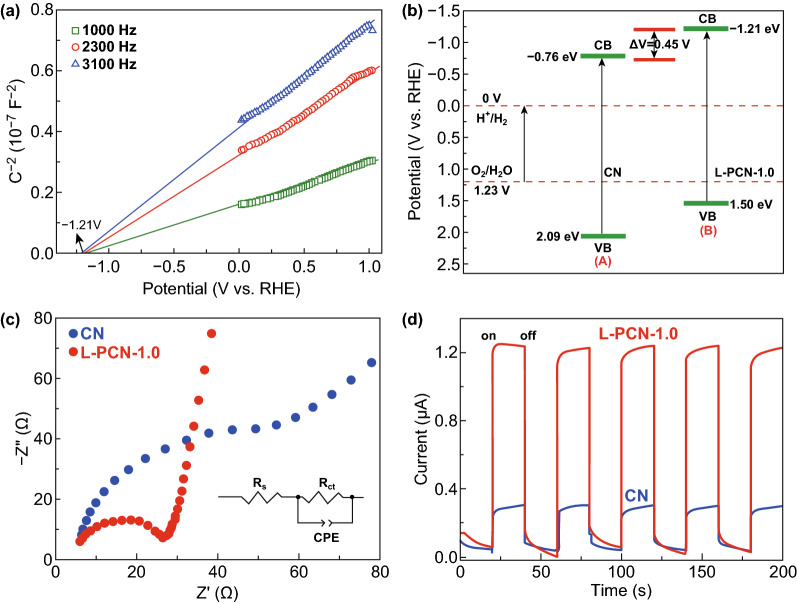
Photoelectrochemical characterization of CN and L-PCN. **a** Mott–Schottky plots with various frequencies of 1.0, 2.3, and 3.1 kHz for L-PCN-1.0 in 0.2 M Na_2_SO_4_ aqueous solution (PH = 6.8). **b** Band structure diagram for CN and L-PCN-1.0. **c** Electrochemical impedance spectroscopy (EIS) Nyquist plots for CN and L-PCN-1.0 obtained in the dark. **d** Transient photocurrents of CN and L-PCN-1.0 in 0.2 M Na_2_SO_4_ aqueous solution

To make sure that the as-prepared L-PCN catalyst is suitable for photocatalytic hydrogen evolution under visible light, the electronic band structure is determined by the analysis of Mott–Schottky plots at different frequencies of 1.0, 2.3, and 3.1 kHz, respectively (Figs. 6a and S12). The positive slope of CN and L-PCN-1.0 originates from the typical n-type characteristic semiconductor, and the flat band potential is ascertained as *x*-intercept in Mott–Schottky plots [24, 50]. In addition, the smaller slope of L-PCN-1.0 in Mott–Schottky plot reflects a higher electron donor density, which is very helpful for improving photocatalytic performance owing to the increased electrical conductivity [10, 28], possibly caused by the unique nanostructure and P-doping effect. The flat band potentials of CN and L-PCN-1.0 are determined to be about − 0.76 and − 1.21 V with the reversible hydrogen electrode (RHE). Compared to bulk CN, the 0.45 V upshift potential for L-PCN-1.0 will lead to a large thermodynamic driving force in photocatalytic hydrogen production. Generally, the flat band potential can be used to approximately estimate the CB position [64, 65]. Herein, combined with the bandgap energy (Fig. 5b), the band structure of the as-prepared photocatalysts can also be obtained (Fig. 6b). Both CN and L-PCN-1.0 satisfied the thermodynamic conditions for photocatalytic water splitting.

Nyquist curve of electrochemical impedance spectroscopy (EIS) and transient photocurrent responses are used to investigate the charge transport behavior of CN and L-PCN catalysts. Generally, the smaller radius of the Nyquist plots corresponds to the faster electron transfer kinetics of the redox reaction and lower charge transfer resistance (*R*_ct_) [66]. The remarkable decrease in Nyquist plot diameter for L-PCN-1.0 (Fig. 6c), L-PCN-0.5, L-PCN-1.5, and L-PCN-2.0 (Fig. S13) catalysts reflects the smaller charge transfer resistance from electrode to electrolyte molecules, originating from the better conductivity of L-PCN, which is in good agreement with the Mott–Schottky tests. Beyond that, transient photocurrent response of CN and L-PCN under visible light illumination (*λ* > 420 nm) in Figs. 6d and S14 shows that all samples exhibited a sensitive photocurrent response during five times on/off irradiation cycles under visible light. Particularly, L-PCN-1.0 shows the largest photocurrent value of about 1.3 μA cm^−2^, which is 4.33 times of bulk CN (0.3 μA cm^−2^), suggesting a high-efficiency charge separation ability for L-PCN-1.0. In addition, the photocurrent responses do not decay with increase in illuminated time, indicating that L-PCN-1.0 catalyst provides a stable quantity of electrons and holes during irradiation.

**Fig. 7:**
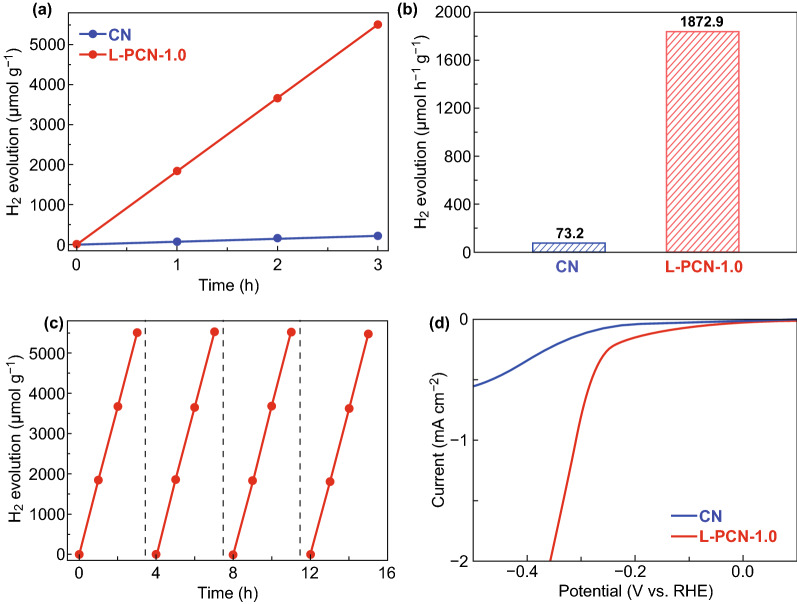
Photo-/electrocatalytic activity characterization of CN and L-PCN. **a** Photocatalytic activity test of H_2_ evolution performance of CN and L-PCN-1.0 with 1 wt% Pt under visible light irradiation (*λ* > 420 nm). **b** H_2_ evolution rate of CN and L-PCN-1.0. **c** Four cycling test of photocatalytic H_2_ evolution of L-PCN-1.0. **d** Linear sweep voltammetry (LSV) plots of CN and L-PCN-1.0 catalysts for HER catalysis in 0.5 M H_2_SO_4_

The photocatalytic activity of CN and L-PCN catalysts is evaluated via hydrogen production from water splitting under visible light irradiation (*λ* > 420 nm). As shown in Fig. 7a, 10.994 μmol for bulk CN and 280.935 μmol H_2_ for L-PCN-1.0 are generated after 3 h visible light irradiation (methyl alcohol as the sacrificial agent and all samples are loading 1% Pt), respectively. Besides that, the hydrogen evolution amount of L-PCN-0.5 (44.94 μmol), L-PCN-1.5 (108.53 μmol), and L-PCN-2.0 (100.65 μmol) samples is also higher than that of over bulk CN (Fig. S15), arising from their relatively high surface area, enhanced visible light absorption, and high charge transport capability (Table S1, Figs. S9 and S13). The average hydrogen production rate (Fig. 7b) for L-PCN-1.0 can be calculated to be 1872.9 μmol h^−1^ g^−1^, which is 25.6 times higher than that of bulk CN (73.2 μmol h^−1^ g^−1^). The apparent quantum yield (AQY) for hydrogen evolution of L-PCN-1.0 under monochromatic light irradiation conditions is estimated to be about 6.93% at 420 ± 15 nm. It is deserved to be mentioned that the catalytic performance of L-PCN-1.0 is better than most reported values of P-doped CN (Table S7). Better yet, L-PCN-1.0 is reused over four cycles without noticeable deactivation of its hydrogen production (Fig. 7c), indicating the high stability of photocatalytic hydrogen yielding, which is in good agreement with the photocurrent results (Fig. 5d). Besides that, the electrocatalytic activity for hydrogen evolution in 0.5 M H_2_SO_4_ medium over bulk CN and L-PCN-1.0 cathodes is investigated in a typical three-electrode cell with reference to the RHE. Obviously, L-PCN-1.0 catalyst shows higher electrocatalytic activity for hydrogen evolution in contrast to bulk CN and other L-PCN with different phosphorus acid added (Fig. S16). Moreover, L-PCN-1.0 also possesses excellent photocatalytic activity to degrade organic pollutants (RhB and BPA) under visible light irradiation (Fig. S17).

**Fig. 8:**
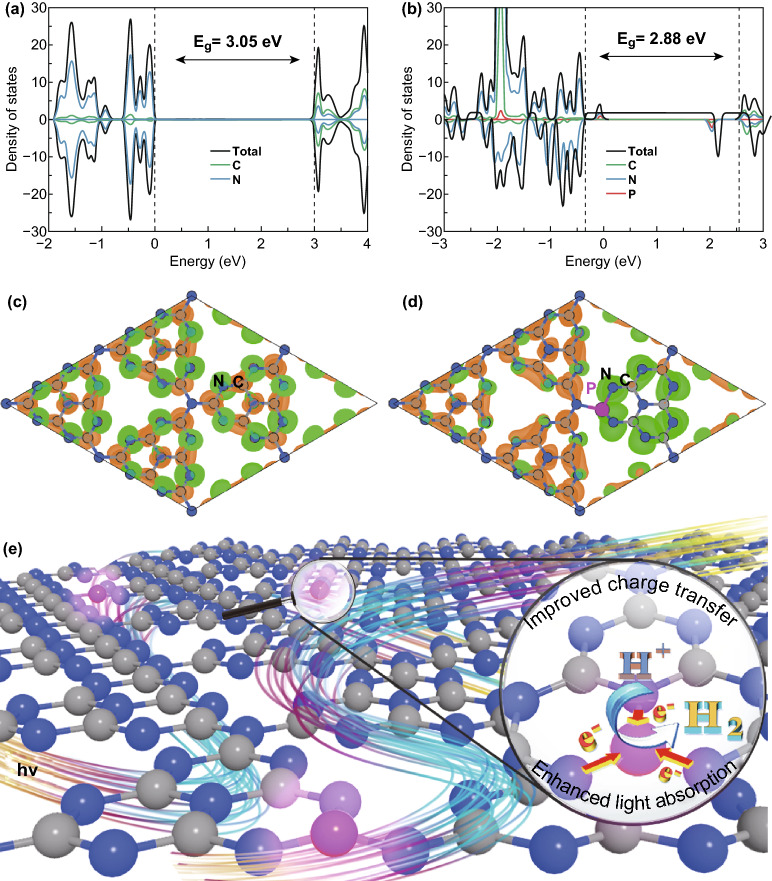
DFT calculations and photocatalytic mechanism of L-PCN. Total density of states and partial density of states for **a** CN and **b** P-doped CN. The electron density distributions of the highest occupied (green) and lowest unoccupied orbit (orange) of **c** CN and **d** P-doped CN with an isovalue of 0.02*e* Å^−3^. **e** Illustration of the photocatalytic process over L-PCN photocatalyst for hydrogen evolution. Gray, blue, and pink spheres represent the C, N, and P atoms. (Color figure online)

To elucidate the fundamental effects of P doping in CN, we performed DFT calculations. According to the experiment results (Fig. 4), the P atoms are introduced in the computational model, in which the P atom replaces the corner carbon atoms (Fig. S18). The density of states projected on each atomic species of pristine CN and P-doped CN is displayed in Fig. 8. The valence band maximum (VBM) of CN is mainly consisted of N orbital, while the conduction band minimum (CBM) is contributed by the C and N orbital together (Fig. 8a). After doping P atom, the interfacial formation energy increases slightly to 12.22 meV Å^−1^ (Fig. S19) compared to pure CN (11.76 meV Å^−1^). As the interlayer distance increases, the interfacial formation energy is also lower than that before doping, suggesting a stronger interlayer binding in P-doped CN. After doping P atoms into the CN, the bandgap decreases from 3.05 to 2.88 eV (Fig. 8b), consistent with the general trend seen in the experiment data above. The P doping does not alter the composition of the band edges, but induces a new mid-gap state above the VBM, leading to the increasing absorption tail of the L-PCN-1.0 in Figs. 5a and S20. Interestingly, we note that the P doping can spatially separate the highest occupied molecular orbital (HOMO) and lowest unoccupied molecular orbital (LUMO). For the pristine CN, the HOMO and LUMO are mainly consisted of N and C orbital, respectively, and distribute on the heptazine units evenly (Fig. 8c). By contrast, the HOMO of P-doped mainly distributes on the heptazine doped with P atom, while the LUMO distributes on other heptazines, as shown in Fig. 8d. It results in an efficient spatial separation of photogenerated carriers, thereby inhibiting their recombination.

Based on the above discussion, it is conceivable that the remarkable photo-/electrocatalytic water splitting activity of the L-PCN-1.0 catalyst can be ascribed to the following factors. The modification of the electronic structure owing to P doping effect promotes more visible light harvesting and provides efficient charge transfer channels between different tri-s-triazine rings (Fig. 8e). Based on a combined analysis of DRS and the Mott–Schottky plots (Figs. 5a, 6a, b), L-PCN-1.0 can absorb more visible light and thus have a higher flat potential and donor density, which promotes the photocatalytic reaction thermodynamically. Additionally, the unique hierarchical self-assembly of well-defined louver-like P-doped CN nanowire arrays exhibits a larger specific surface area (121 m^2^ g^−1^, Fig. 3a), more boundaries, and active sites exposed, as well as higher mass transport in hydrogen evolution process originated from the clearance spaces among CN nanowires (SEM image, Fig. 2b), resulting in excellent hydrogen production performance. Furthermore, L-PCN-1.0 possesses prolonged separation life of photogenerated carriers. The sharp decreased PL spectra (Fig. 5c), the lengthened decay time of charge carriers (Fig. 5d), smaller diameter of the EIS Nyquist curve, and stronger response of the transient photocurrent plots (Fig. 6c, d) all revealed the markedly improved electromobility efficiency of charge carriers; these diversified active elements pave the way for highly synergistic improving the hydrogen evolution reaction under visible light irradiation.

## Conclusions

In summary, we show a facile yet effective route toward precisely manipulated synthesis of well-defined louver-like P-doped carbon nitride nanowire arrays via a hierarchical supramolecular self-assembly method by regulating the hydrogen bond. The controlled realization of self-assembly P-doped carbon nitride nanowire arrays with appropriate separation and spatial location of each CN nanowire integrates full potential of assembled nanostructures by virtue of the collective properties of single CN nanowire as well as ultrastability owing to the well-defined frame. Owing to the unique louver-like nanostructure with optimized optical and electronic structure characters, L-PCN nanowire arrays demonstrate its efficient and hyperstatic visible light-driven water splitting hydrogen evolution activity of 1872.9 μmol h^−1^ g^−1^ and an apparent quantum efficiency of 6.93% at 420 ± 15 nm. This hierarchical self-assembly approach releases the potential of single nanostructures self-assembly into diversified nanostructure arrays and facilitates the manufacturing of nanomaterials with diverse shapes and functional properties.

## Electronic supplementary material

Below is the link to the electronic supplementary material.

Supplementary material 1 (PDF 1709 kb)
