# Supplementary material for: Hierarchical Self-assembly of Well-Defined Louver-Like P-Doped Carbon Nitride Nanowire Arrays with Highly Efficient Hydrogen Evolution
Source: Nanomicro Lett. 2020 Feb 17;12:52. doi: 10.1007/s40820-020-0399-1 (PMC7770876; doi:10.1007/s40820-020-0399-1)
Supplement: Supplementary file 1 — Supplementary material 1 (PDF 1709 kb) [file 40820_2020_399_MOESM1_ESM.pdf]

Supporting Information for

Hierarchical Self-assembly of Well-defined Louver-like P-doped Carbon Nitride Nanowire Arrays with Highly Efficient Hydrogen Evolution

Bo Li[1], Yuan Si[1], Qian Fang[1], Ying Shi[2], Wei-Qing Huang[1,]*, Wangyu Hu[1,]*, Anlian Pan[1], Xiaoxing Fan[3], Gui-Fang Huang[1,]*

[1]Department of Applied Physics, College of Physics and Electronics, and College of Materials Science and Engineering, and State key laboratory of chemo/biosensing and chemometrics, College of Chemistry and Chemical Engineering, Hunan University, Changsha 410082, People's Republic of China

[2]Department of Physics and Tsinghua-Foxconn Nanotechnology Research Center, Tsinghua University, Beijing 100084, People's Republic of China

[3]College of Physics, Liaoning University, Shenyang 110036, People's Republic of China

*Corresponding authors. E-mail: wqhuang@hnu.edu.cn (Wei-Qing Huang); wyuhu@hnu.edu.cn (Wangyu Hu); gfhuang@hnu.edu.cn (Gui-Fang Huang)

Supplementary Figures and Tables

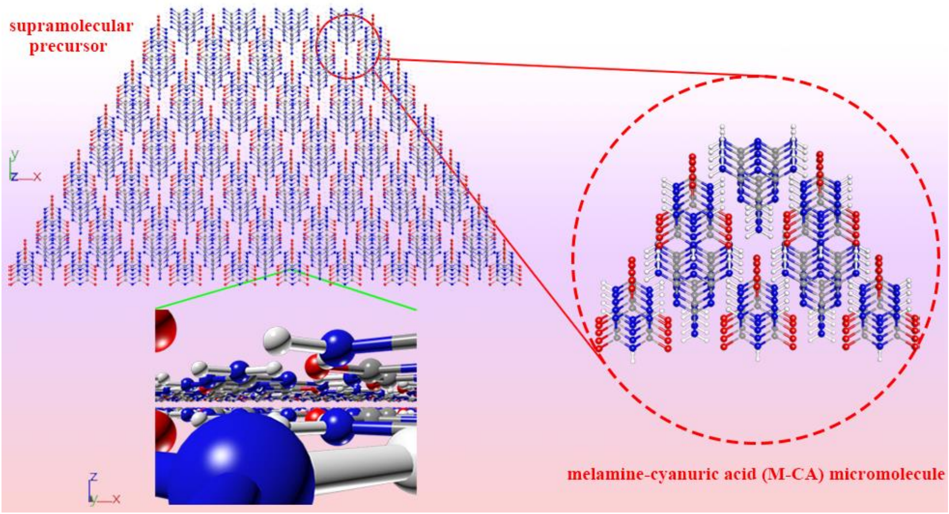

Fig. S1 Schematic illustration of the hierarchical self-assembly strategy based on the hydrogen bonding interaction. Adjacent melamines and cyanuric acid are connected crossly by the hydrogen bonds and stacked in a perpendicular direction to the triazines using π-π interaction to form M-CA micromolecule, finally yield a quadrangular-like supramolecular precursor.

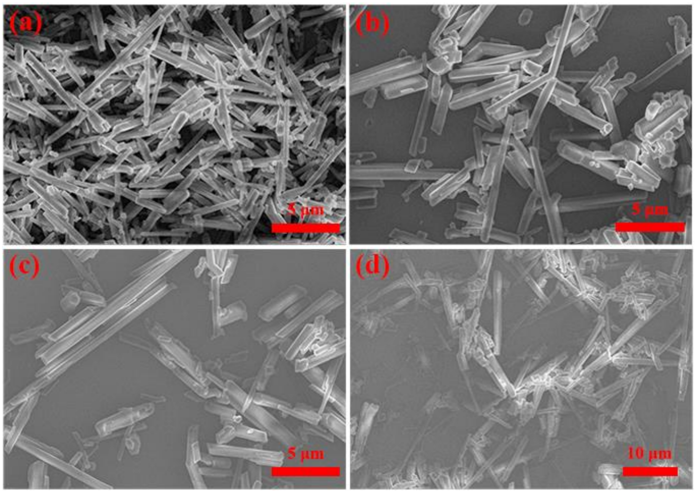

Fig. S2 SEM images of as-prepared supramolecular precursors **a**) CN and **b-d**) L-PCN-0.5, L-PCN-1.5 and L-PCN-2.0

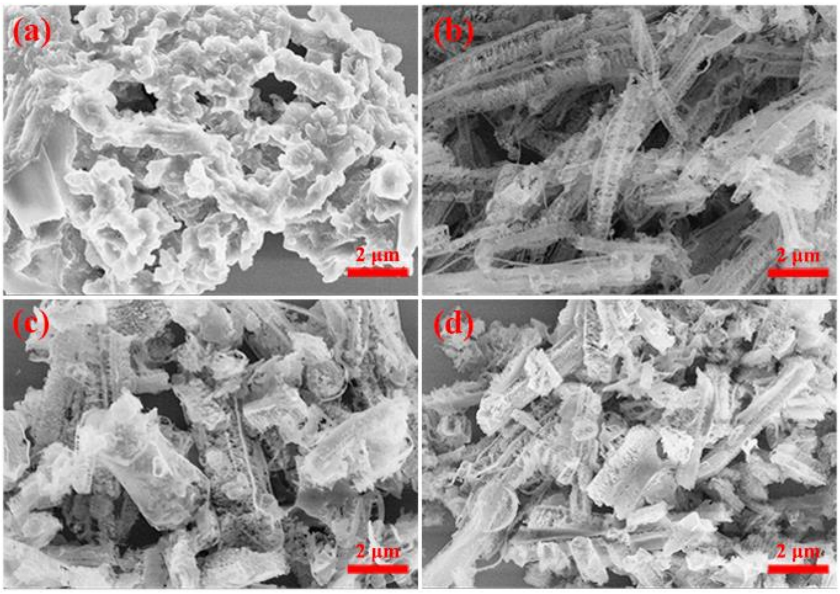

Fig. S3 SEM images of **a**) CN and **b-d**) L-PCN-0.5, L-PCN-1.5 and L-PCN-2.0

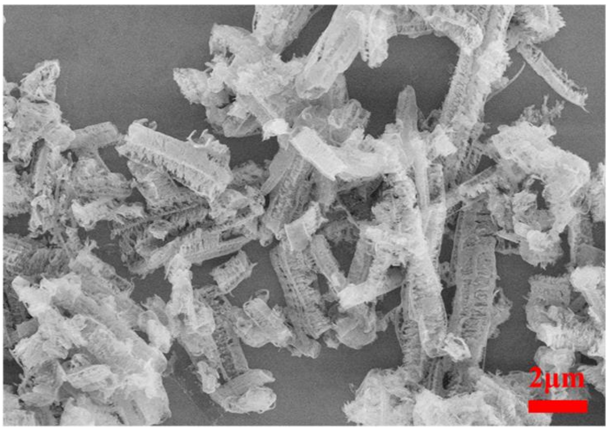

Fig. S4 SEM images of L-PCN-1.0

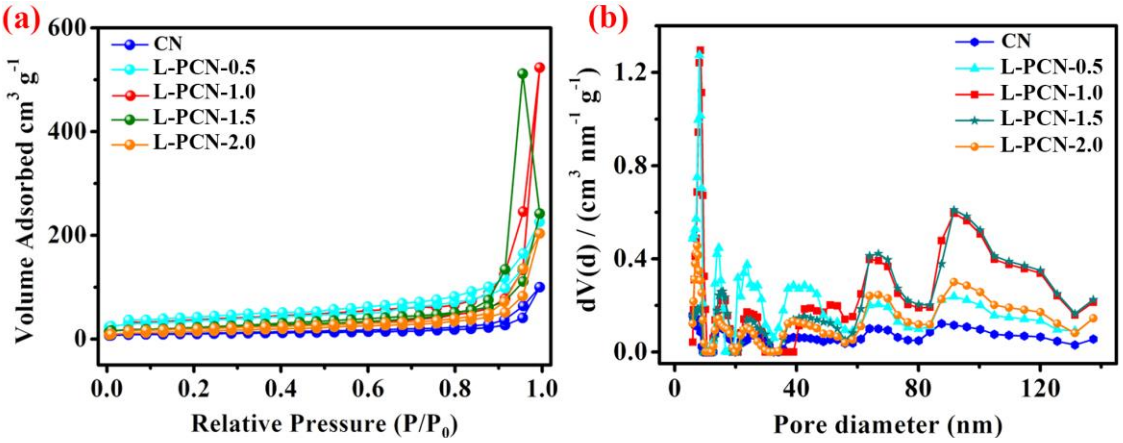

Fig. S5 a) Nitrogen adsorption-desorption isotherms and **b**) corresponding pore size distribution curves of CN, L-PCN-0.5, L-PCN-1.0, L-PCN-1.5 and L-PCN-2.0

Table S1 Summary of pore volumes and specific surface area of CN, L-PCN-0.5, L-PCN-1.0, L-PCN-1.5 and L-PCN-2.0

Samples	CN	L-PCN-0.5	L-PCN-1.0	L-PCN-1.5	L-PCN-2.0
Pore volume (cm^3 g^{-1})	0.14	0.32	0.8	0.78	0.31
surface areas (m^2 g^{-1})	33	68	121	70	53

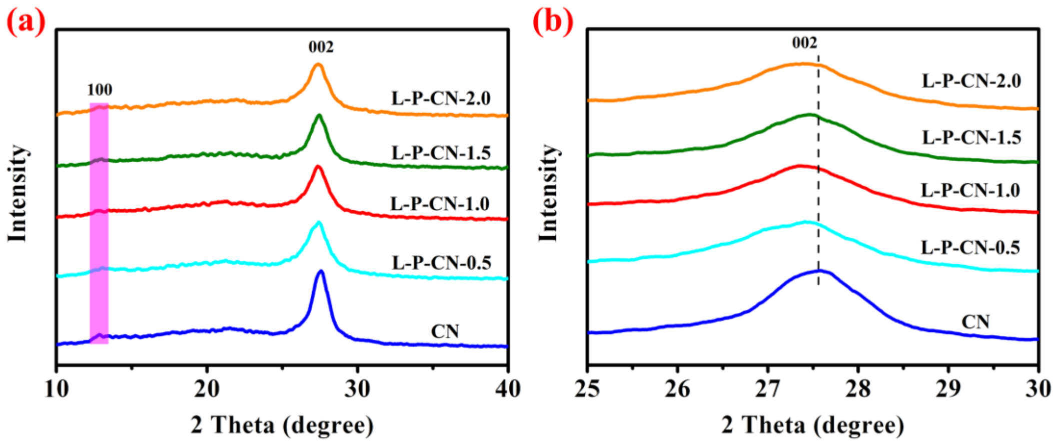

Fig. S6 XRD patterns of **a, b**) CN, L-PCN-0.5, L-PCN-1.0, L-PCN-1.5 and L-PCN-2.0

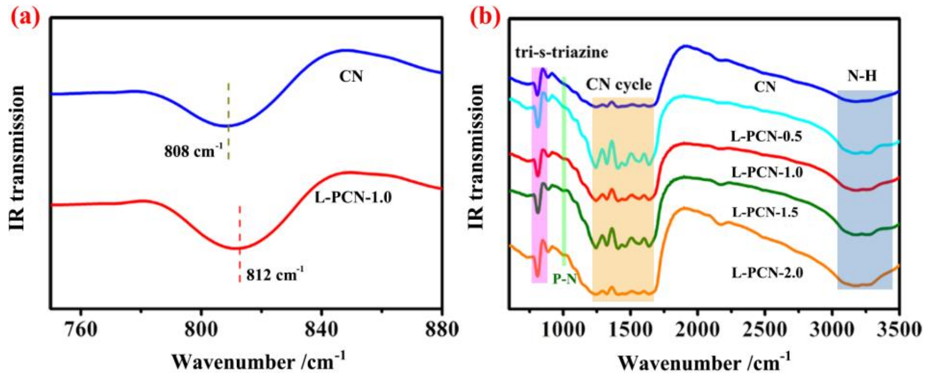

Fig. S7 a) High resolution FT-IR spectra of CN and L-PCN-1.0. **b**) FT-IR spectra of CN, L-PCN-0.5, L-PCN-1.0, L-PCN-1.5 and L-PCN-2.0 at room temperature

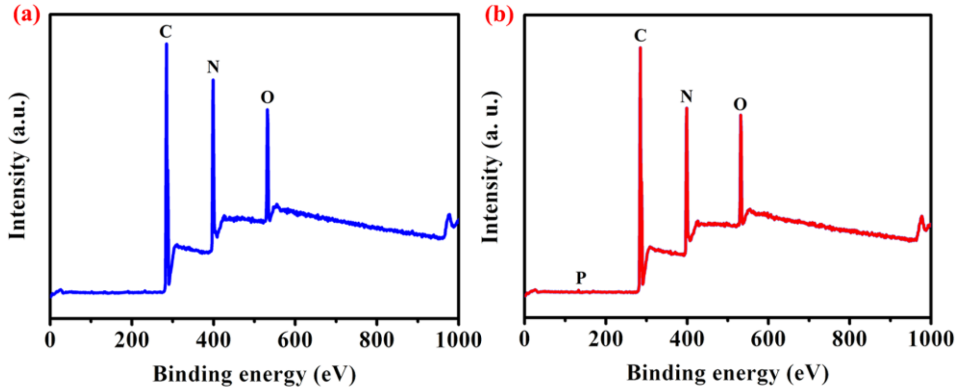

Fig. S8 XPS survey spectra of **a**) CN and **b**) L-PCN-1.0

Table S2 Summarized C 1s and N1s data for CN and L-PCN-1.0

Samples	Atomic compositions (%)				
	$C-NH_x/C$	N-C=N/C	N_2C/N	N_3C/N	$C-NH_x/N$
CN	28.51	71.49	75.35	15.92	8.73
L-PCN-1.0	28.58	71.42	76.58	13.92	9.50

Table S3 Summarized C 1s and N1s data for CN and L-PCN-1.0

Samples	Binding energy (eV)					
	C-C	$C-NH_x$	N-C=N	N_2C	N_3C	NH_x
CN	284.8	286.5	288.3	398.5	399.6	400.8
L-PCN-1.0	284.8	286.4	288.1	398.6	399.9	401.1

Table S4 Surface relative element content of CN and L-PCN-1.0 from XPS characterizes

Samples	Atomic compositions (%)		
	C	N	P
CN	45.06	54.94	-
L-PCN-1.0	45.54	54.21	0.25

Table S5 EDS analysis of CN, L-PCN-0.5, L-PCN-1.0, L-PCN-1.5 and L-PCN-2.0

Samples	Atomic percent (%)			
	C	N	P	C/N
CN	31.47	68.53	-	0.459
L-PCN-0.5	29.59	70.14	0.27	0.422
L-PCN-1.0	31.25	68.47	0.28	0.456
L-PCN-1.5	31.98	67.49	0.53	0.472
L-PCN-2.0	33.43	65.40	1.17	0.511

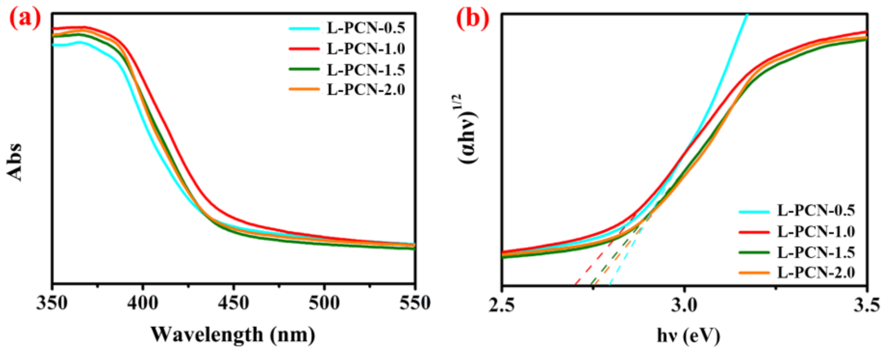

Fig. S9 a) UV-DRS spectra and **b**) plots of transformed Kubelka-Munk function versus photon energy for L-PCN-0.5, L-PCN-1.0, L-PCN-1.5 and L-PCN-2.0

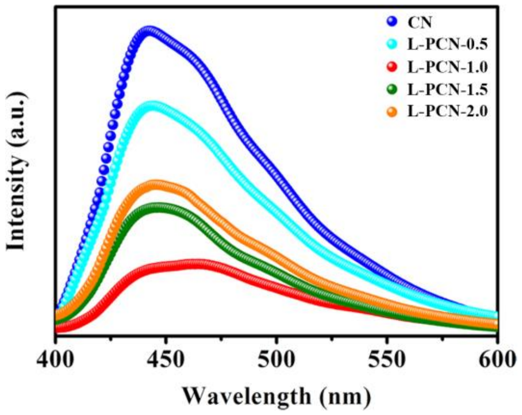

Fig. S10 Photoluminescence (PL) emission spectra of CN, L-PCN-0.5, L-PCN-1.0, L-PCN-1.5 and L-PCN-2.0 (with the excitation wavelength of 350 nm at room temperature)

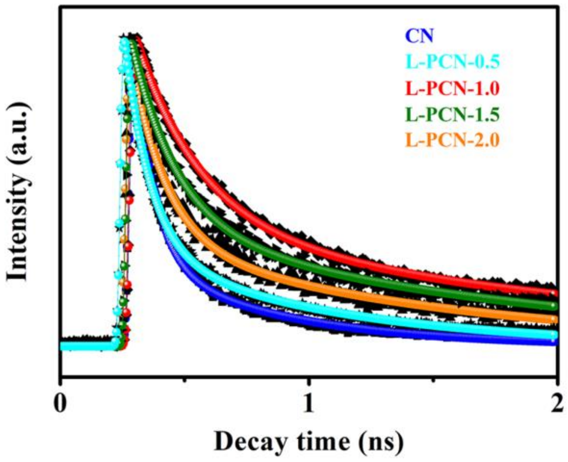

Fig. S11 Time-resolved fluorescence decay spectra of CN, L-PCN-0.5, L-PCN-1.0, L-PCN-1.5 and L-PCN-2.0. These spectra were recorded with the excitation of 400 nm from a picosecond pulsed light-emitting diode at room temperature

Table S6 Summary of time-resolved fluorescence decay time (τ) and their relative amplitude (A) in the CN, L-PCN-0.5, L-PCN-1.0, L-PCN-1.5, and L-PCN-2.0

Samples	Decay time (ns)		Relative amplitude (%)		Average life Time (< τ >, ns)
	τ_1	τ_2	A_1	A_2	
CN	0.08	0.58	75.85	24.15	0.41
L-PCN-0.5	0.14	1.21	66.87	33.13	1.01
L-PCN-1.0	0.26	1.95	62.18	37.82	1.66
L-PCN-1.5	0.18	1.53	63.02	36.98	1.31
L-PCN-2.0	0.19	1.48	62.39	37.61	1.26

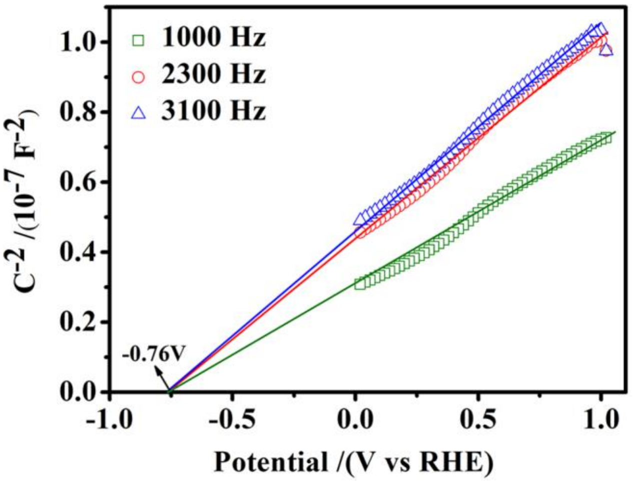

Fig. S12 Mott-Schottky plots with various frequencies of 1.0, 2.3 and 3.1 KHz for bulk CN with refer to the Reversible Hydrogen Electrode (RHE)

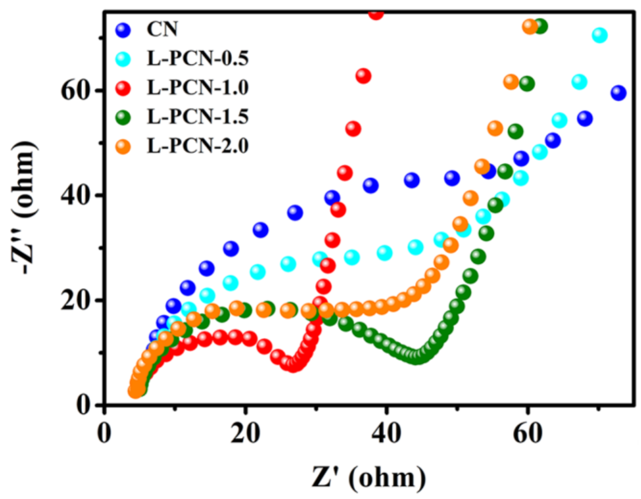

Fig. S13 Nyquist curve of electrochemical impedance spectroscopy (EIS) for CN, L-PCN-0.5, L-PCN-1.0, L-PCN-1.5 and L-PCN-2.0 in 0.2 м Na_2SO_4 aqueous solution (PH = 6.8) under dark environment

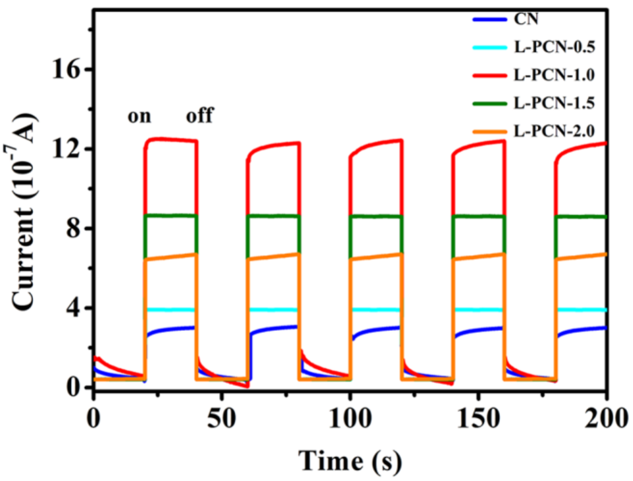

Fig. S14 Transient photocurrent responses plots of CN, L-PCN-0.5, L-PCN-1.0, L-PCN-1.5 and L-PCN-2.0 in 0.2 м Na_2SO_4 aqueous solution (PH = 6.8)

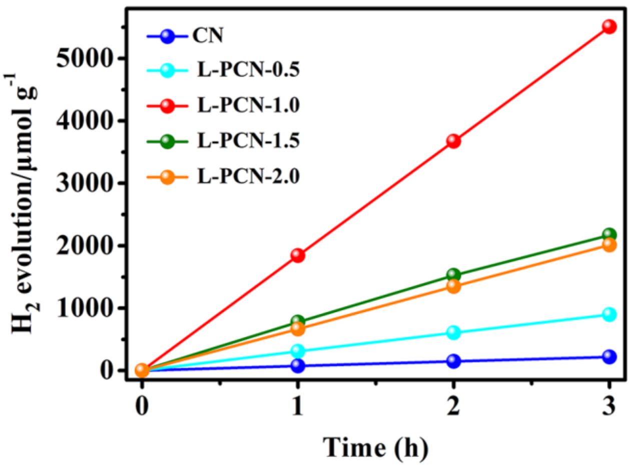

Fig. S15 Photocatalytic activity test of hydrogen evolution performance of CN, L-PCN-0.5, L-PCN-1.0, L-PCN-1.5 and L-PCN-2.0 with 1 wt% Pt under visible light irradiation (λ>420 nm)

Table S7 Comparison of the HER performance of L-PCN with previously reported HER photocatalysts

Catalysts	HER rate μmol h^{-1}	Amount of catalyst (mg)	Light source	AQY	Ref.
**L-PCN**	**93.6**	**50 (1% Pt)**	**λ>420 nm**	**6.93%**	**This work**
P-TCN	67	100 (1% Pt)	λ>420 nm	5.68%	S1
B/P-CNNs	602.6	50 (1% Pt)	λ>400 nm	7.55%	S2
P-doped CN	50.6	100 (3% Pt)	λ>420 nm	N/A	S3
PCN-S	79.8	50 (1% Pt)	λ>420 nm	3.56%	S4
P-CN	3.39	30 (N/A)	N/A	N/A	S5
P-CN	104.1	50 (3% Pt)	N/A	N/A	S6
PCNT	50.7	100 (1% Pt)	λ>420 nm	N/A	S7
PCNT	101	50 (3% Pt)	λ>420 nm	4.32%	S8
P@PCN	94.1	100 (1% Pt)	λ>420 nm	N/A	S9
PCN	54.1	30 (3% Pt)	λ>400 nm	8.96%	S10
PCN NS	256.4	50 (3% Pt)	λ>400 nm	N/A	S11
CPCN	74.6	50 (1% Pt)	λ>420 nm	2.14%	S12
PCN	130.54	50 (1% Pt)	λ>420 nm	8.5%	S13
A-CN	71	10 (1% Pt)	λ>420 nm	7.4	S14

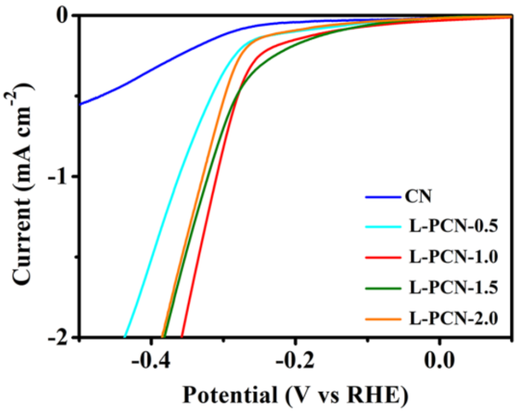

Fig. S16 Linear sweep voltammetry (LSV) plots for CN, L-PCN-0.5, L-PCN-1.0, L-PCN-1.5 and L-PCN-2.0 in 0.5 M H_2SO_4 aqueous solution with refer to the reversible hydrogen electrode (RHE)

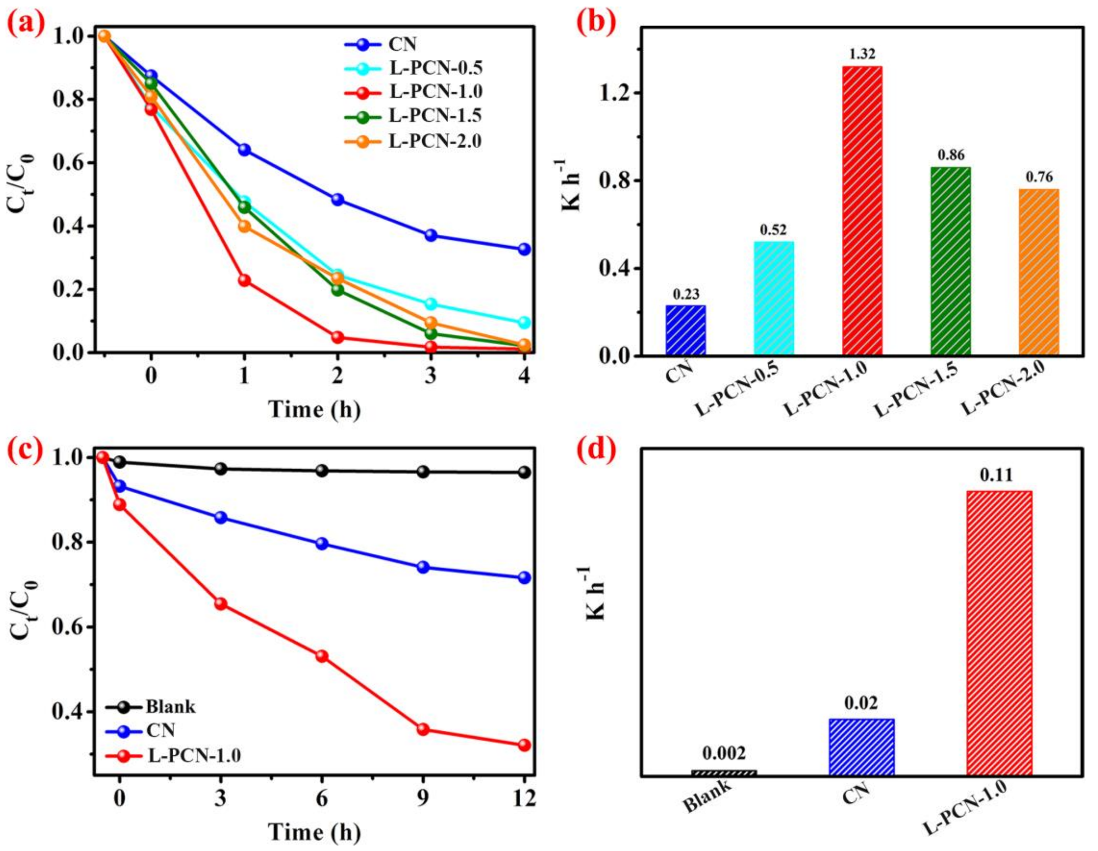

Fig. S17 a) Photodegradation behaviors and **b**) degradation rate constant k (h^{-1}) of Rhodamine B (RhB, 10 mg L^{-1}) on bulk CN, L-PCN-0.5, L-PCN-1.0, L-PCN-1.5 and L-PCN-2.0 under visible-light irradiation (300 W halogen lamp). **c**) Photodegradation behaviors and d) degradation rate constant k (h^{-1}) of bisphenol A (BPA, 10 mg L^{-1}) on bulk CN and L-PCN-1.0 under visible-light irradiation (300 W halogen lamp).

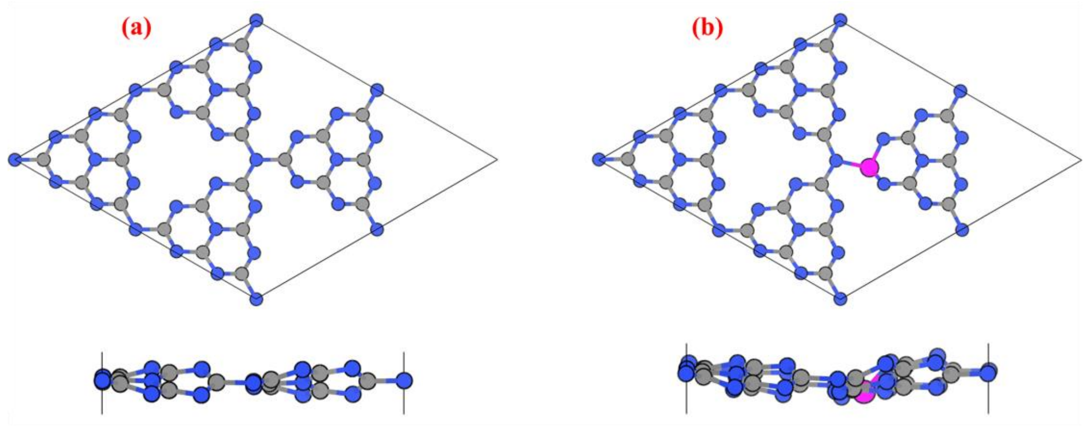

Fig. S18 Top and side views of (**a**) CN and (**b**) P-doped CN. Grey, blue and pink circles represent the carbon, nitrogen, and phosphorous

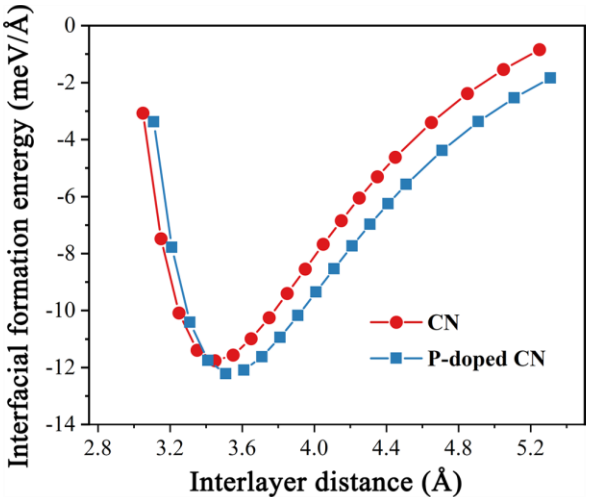

Fig. S19 The evolution of the interfacial formation energy of CN and P-doped CN as a function of the interlayer distance

The evolution of the interfacial formation energy of CN and P-doped CN as a function of the interlayer distance. The interfacial formation energy is defined as: $E_{if} = \frac{1}{S}(E_{bilayer} - 2E_{monolayer})$, where S is the area of the interface; $E_{bilayer}$ and $E_{monolayer}$ represent the total energies of the bilayer and monolayer, respectively. This metric can quantitatively evaluate the intrinsic stability of system, as well as the type of interaction, because its value is independent to size of calculated cell. As displayed in Fig. S19, The interlayer distance and interfacial formation energy of CN are 3.45 Å and 11.76 meV/Å respectively, which are similar to the computed values of typical vdW materials, suggesting that the vdW interaction (non-covalent interactions) dominate the interlayer binding. After doping P atom, the interfacial formation energy is increase slightly to 12.22 meV/Å. As the interlayer distance increases, the interfacial formation energy is also lower than it before doping, suggesting a stronger interlayer binding in P-doped CN, which is attribute the enhancement of the electrostatic interaction. Due to this stronger interlayer binding, the experimental CN nanowire arrays with well-defined louver-like nanostructure can be maintain after thermal treatment with 500 degree centigrade, 4h.

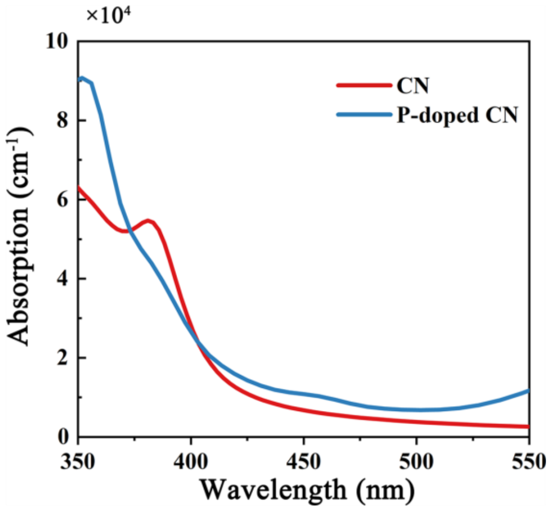

Fig. S20 Computational absorption spectrums of CN and P-doped CN

Computational Parameters

SYSTEM = P-doped CN

ENCUT = 500

ISTART = 0; ICHARG = 2

ISIF=2

IBRION=2

NSW=500

NELM=60

NELMIN=4

ISMEAR = 0

SIGMA=0.1

ALGO=Fast

PREC = Normal

EDIFF=1E-6

EDIFFG=-0.01

IVDW=10

LREAL = Auto

LWAVE=.FALSE.

LCHARG=.FALSE.

CONTCAR

1.0

14.2466001511	0.0000000000	0.0000000000
-7.1233000755	12.3379176484	0.0000000000
0.0000000000	0.0000000000	13.0000000000

C	N	P
23	32	1

Direct

0.058060002	0.282339007	0.527585983

0.882341027	0.939705014	0.524528980
0.062778004	0.123772003	0.510141015
0.220746994	0.280993015	0.471302003
0.214233994	0.447833002	0.466352999
0.566444993	0.117071003	0.522183001
0.879733026	0.437335014	0.479130000
0.560833991	0.272493988	0.540513992
0.723657012	0.272035003	0.479690999
0.720852971	0.439319015	0.468497992
0.064078003	0.943497002	0.503584981
0.059354998	0.620274007	0.500750005
0.383282006	0.939037979	0.528301001
0.059645999	0.781134009	0.483213007
0.222812995	0.775394976	0.532828987
0.226703003	0.947575986	0.518848002
0.054644000	0.439678013	0.499401987
0.558486998	0.624632001	0.480789006
0.562092006	0.940585017	0.510873020
0.561120987	0.785125971	0.470180988
0.722886026	0.775201976	0.516847014
0.724007010	0.945173979	0.527698994
0.374278992	0.451379001	0.463506997
0.002393000	0.001792000	0.513226986
0.152423993	0.492733985	0.456236005
0.013049000	0.175611004	0.547291994
0.328803986	0.341935992	0.458101004
0.008711000	0.340811998	0.543954015
0.321610987	0.505895972	0.458068997
0.163554996	0.172242001	0.469992995
0.164198995	0.336903006	0.488346994
0.502152026	0.997403979	0.522441030
0.665087998	0.486196011	0.439819992
0.520695984	0.168402001	0.567618012
0.831530988	0.328310013	0.466500014
0.504459977	0.323702991	0.549263000

0.828696012	0.493185997	0.471688002
0.663405001	0.162841007	0.475713998
0.667405009	0.327805012	0.497099996
0.996279001	0.498876005	0.495968014
0.169444993	0.998787999	0.528129995
0.009132000	0.673354983	0.469420999
0.331021011	0.831066012	0.547384024
0.010402000	0.840090990	0.470510006
0.335034013	0.998628020	0.516487002
0.161360994	0.666356981	0.537769020
0.169565007	0.834407985	0.511402011
0.489345014	0.506676972	0.476047993
0.664268970	0.991783023	0.546487987
0.511059999	0.679629982	0.447912991
0.830940008	0.830088973	0.530578971
0.511691988	0.843964994	0.463723987
0.832342982	0.997662008	0.531075001
0.660336971	0.665884972	0.516737998
0.669475973	0.835079014	0.502822995
0.533329010	0.412330002	0.456196994

Supplementary References

[S1]S. Guo, Z. Deng, M. Li, B. Jiang, C. Tian, Q. Pan, H. Fu, Phosphorus-doped carbon nitride tubes with a layered micro-nanostructure for enhanced visible-light photocatalytic hydrogen evolution. Angew. Chem. Int. Ed. **55**(5), 1830-1834 (2016). https://doi.org/10.1002/anie.201508505

[S2]B. Li, Y. Si, B.X. Zhou, Q. Fang, Y.Y. Li et al., Doping-induced hydrogen-bond engineering in polymeric carbon nitride to significantly boost the photocatalytic H_2 evolution performance. ACS Appl. Mater. Interfaces **11**(19), 17341-17349 (2019). https://doi.org/10.1021/acsami.8b22366

[S3]Y. Zhou, L. Zhang, J. Liu, X. Fan, B. Wang et al., Brand new P-doped g-C_3N_4: Enhanced photocatalytic activity for H_2 evolution and Rhodamine B degradation under visible light. J. Mater. Chem. A **3**(7), 3862-3867 (2015). https://doi.org/10.1039/c4ta05292g

[S4]R.J. Ran, T.Y. Ma, G. Gao, X. Du, S. Qiao, Porous P-doped graphitic carbon nitride nanosheets for synergistically enhanced visible-light photocatalytic H_2 production. Energy Environ. Sci. **8**(12), 3708-3717 (2015). https://doi.org/10.1039/c5ee02650d

[S5]B. Liu, L.Q. Ye, R. Wang, J.F. Yang, Y.X. Zhang, R. Guan, L.H. Tian, X.B. Chen,

Phosphorus-doped graphitic carbon nitride nanotubes with amino-rich surface for efficient CO_2 capture, enhanced photocatalytic activity, and product selectivity. ACS Appl. Mater. Interfaces **10**(4), 4001-4009 (2018). https://doi.org/10.1021/acsami.7b17503

[S6]Y.P. Zhu, T.Z. Ren, Z.Y. Yuana, Mesoporous phosphorus-doped g-C_3N_4 nanostructured flowers with superior photocatalytic hydrogen evolution performance. ACS Appl. Mater. Interfaces **7**(30), 16850-16856 (2015). https://doi.org/10.1021/acsami.5b04947

[S7]S.E. Guo, Y.Q. Tang, Y. Xie, C.G. Tian, Q.M. Feng, W. Zhou, B.J. Jiang, P-doped tubular g-C_3N_4 with surface carbon defects: Universal synthesis and enhanced visible-light photocatalytic hydrogen production. Appl. Catal. B: Environ. **218**, 664-671 (2017). https://doi.org/10.1016/j.apcatb.2017.07.022

[S8]M. Wu, J. Zhang, B.B. He, H.W. Wang, R. Wang, Y.S. Gong, In-situ construction of coral-like porous P-doped g-C_3N_4 tubes with hybrid 1D/2D architecture and high efficient photocatalytic hydrogen evolution. Appl. Catal. B: Environ. **241**, 159-166 (2019). https://doi.org/10.1016/j.apcatb.2018.09.037

[S9]J.J. Feng, D.K. Zhang, H.P. Zhou, M.Y. Pi, X.D. Wang, S.J. Chen, Coupling P nanostructures with P-doped g-C_3N_4 as efficient visible light photocatalysts for H_2 evolution and rhb degradation. ACS Sustain. Chem. Eng. **6**(5), 6342-6349 (2018). https://doi.org/10.1021/acssuschemeng.8b00140

[S10] X. Tian, Y.J. Sun, J.Y. He, X.J. Wang, J. Zhao, S.Z. Qiao, F.T. Li, Surface P atom grafting of g-C_3N_4 for improved local spatial charge separation and enhanced photocatalytic H_2 production. J. Mater. Chem. A **7**(13), 7628-7635 (2019). https://doi.org/10.1039/c9ta00129h

[S11] H.Y. Yang, Y.M. Zhou, Y.Y. Wang, S.C. Hu, B.B. Wang et al., Three-dimensional flower-like phosphorus-doped g-C_3N_{4-} with a high surface area for visible-light photocatalytic hydrogen evolution. J. Mater. Chem. A **6**(34), 16485-16494 (2018). https://doi.org/10.1039/c8ta05723k

[S12] H. Wang, B. Wang, Y.R. Bian, L.M. Dai, Enhancing photocatalytic activity of graphitic carbon nitride by codoping with P and C for efficient hydrogen generation. ACS Appl. Mater. Interfaces **9**(26), 21730-21737 (2017). https://doi.org/10.1021/acsami.7b02445

[S13] X.X. Fang, L.B. Ma, K. Liang, S.J. Zhao, Y.F. Jiang et al., The doping of phosphorus atoms into graphitic carbon nitride for highly enhanced photocatalytic hydrogen evolution. J. Mater. Chem. A **7**(18), 11506-11512 (2019). https://doi.org/10.1039/c9ta01646e

[S14] C. Zhao, Q. Li, Y. Xie, L.P. Zhang, X.D. Xiao et al., Three-dimensional assemblies of carbon nitride tubes as nanoreactors for enhanced photocatalytic hydrogen production. J. Mater. Chem. A **8**(1), 305-312 (2020). https://doi.org/10.1039/c9ta10688j
